# Personality predicts foraging site fidelity and trip repeatability in a marine predator

**DOI:** 10.1111/1365-2656.13106

**Published:** 2019-10-18

**Authors:** Stephanie M. Harris, Sébastien Descamps, Lynne U. Sneddon, Philip Bertrand, Olivier Chastel, Samantha C. Patrick

**Affiliations:** ^1^ School of Environmental Sciences University of Liverpool Liverpool UK; ^2^ Norwegian Polar Institute, Fram Centre Tromsø Norway; ^3^ Institute of Integrative Biology University of Liverpool Liverpool UK; ^4^ Department of Biology and Center for Northern Studies University of Quebec Rimouski QC Canada; ^5^ Centre d'Etudes Biologiques de Chizé (CEBC) UMR 7372 CNRS Université de La Rochelle Villiers‐en‐Bois France

**Keywords:** biologging, boldness, foraging niche width, foraging specialization, marine vertebrate, movement ecology, personality, site fidelity

## Abstract

1. Animal populations are often comprised of both foraging specialists and generalists. For instance, some individuals show higher foraging site fidelity (spatial specialization) than others. Such individual differences in degree of specialization can persist over time‐scales of months or even years in long‐lived animals, but the mechanisms leading to these different individual strategies are not fully understood.
2. There is accumulating evidence that individual variation in foraging behaviour is shaped by animal personality traits, such as boldness. Despite this, the potential for boldness to drive differences in the degree of specialization is unknown.
3. In this study, we used novel object tests to measure boldness in black‐legged kittiwakes (*Rissa tridactyla*) breeding at four colonies in Svalbard and deployed GPS loggers to examine their at‐sea foraging behaviour. We estimated the repeatability of foraging trips and used a hidden Markov model to identify locations of foraging sites in order to quantify individual foraging site fidelity.
4. Across the breeding season, bolder birds were more repeatable than shy individuals in the distance and range of their foraging trips, and during the incubation period (but not chick rearing), bolder individuals were more site‐faithful. Birds exhibited these differences while showing high spatial similarity in foraging areas, indicating that site selection was not driven by personality‐dependent spatial partitioning.
5. We instead suggest that a relationship between boldness and site fidelity may be driven by differences in behavioural flexibility between bold and shy individuals. Together, these results provide a potential mechanism by which widely reported individual differences in foraging specialization may emerge.

## Graphical abstract

This paper reports the influence of animal personality on foraging specializations in black‐legged kittiwakes, finding that bolder birds show higher repeatability in their foraging behaviour and, during incubation, higher foraging site fidelity. The results indicate that bold individuals are more highly specialized in their foraging behaviour than their shy conspecifics.

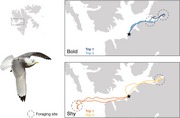

## 1 INTRODUCTION

Among‐individual differences often comprise the majority of a population's variation in behaviour (Araújo, Bolnick, & Layman, 2011; Bolnick et al., 2003; Dall, Bell, Bolnick, & Ratnieks, 2012). Individual foraging specializations are a particularly widespread example, whereby individuals utilize only a subset of the population foraging niche (Bolnick et al., 2003). Foraging site fidelity is a common type of behavioural specialization whereby individuals show spatial consistency in their foraging behaviour, repeatedly visiting the same locations (Baylis, Page, McKenzie, & Goldsworthy, 2012; Hillen, Kiefer, & Veith, 2009; Wakefield et al., 2015). At the population level, site fidelity is thought to result from intraspecific competition for resources (Bolnick et al., 2003), but populations are often comprised of individuals of varying levels of site fidelity, resulting in the coexistence of behavioural specialists and generalists (Arthur et al., 2015; Patrick & Weimerskirch, 2017; Wakefield et al., 2015; Wilson & Yoshimura, 1994). However, while there is increasing evidence of the existence of such differences, the individual‐level drivers of site fidelity are poorly understood. Individual differences in site fidelity are often attributed to age‐ or sex‐related differences (Durell, 2007; Phillips, Silk, Phalan, Catry, & Croxall, 2004; Votier et al., 2017), but in many systems, individual variation in site fidelity remains even once age and sex are accounted for (Bolnick et al., 2003; Votier et al., 2017; Woo, Elliott, Davidson, Gaston, & Davoren, 2008).

Specialized foraging behaviour may be optimal when resource predictability is high, such that individual differences in site fidelity can emerge as an artefact of spatial partitioning if individuals use foraging areas differing in resource predictability (Barraquand & Benhamou, 2008; Switzer, 1993). However, individuals may maintain their level of specialization over time‐scales greater than the persistence of resource patches (Patrick & Weimerskirch, 2017; Wakefield et al., 2015), suggesting that individuals can differ intrinsically in degree of specialization. While foraging differences have been attributed to morphological (Camprasse, Cherel, Bustamante, Arnould, & Bost, 2017; van de Pol, Brouwer, Ens, Oosterbeek, & Tinbergen, 2010) and physiological (Bearhop, Adams, Waldron, Fuller, & Macleod, 2004; Watanabe, 2006) variation, significantly less attention has been paid to the influence of individual behavioural variation or personality differences. Animal personalities are individual differences in behavioural phenotypes, typically measured on behavioural axes, that are consistent over time or context (Gosling, 2001; Réale et al., 2010). The bold–shy personality axis has been linked to various aspects of foraging behaviour, particularly in a spatial context (Patrick & Weimerskirch, 2014; Spiegel, Leu, Sih, Godfrey, & Bull, 2015; Verbeek, Drent, & Wiepkema, 1994). For example, bold and shy individuals have been found to forage over different spatial scales (Patrick & Weimerskirch, 2014; Spiegel et al., 2015) and use different levels of search intensity (van Overveld & Matthysen, 2010; Spiegel, Leu, Bull, & Sih, 2017). Links between boldness and exploration, another commonly studied personality trait which measures space use, are also predicted by the pace‐of‐life syndrome hypothesis (Réale et al., 2010). Cumulatively, theory and empirical findings suggest boldness has high potential to promote differences in foraging behaviour, but to our knowledge, no study has examined the relationship between boldness and foraging site fidelity.

As site fidelity has not been incorporated into the personality research framework, there is no unified prediction regarding the relationship between site fidelity and boldness. However, some evidence does suggest that bolder individuals may be more behaviourally specialized. Bold animals generally exhibit inflexible, routine‐like behavioural tendencies, while shy individuals show greater flexibility, adapting behaviour to prevailing conditions (Benus, Daas, Koolhaas, & Oortmerssen, 1990; Coppens, De Boer, & Koolhaas, 2010; Koolhaas et al., 1999; Wolf, van Doorn, & Weissing, 2008). Consequently, bold individuals may be more site‐faithful as they use the same foraging routes and the same foraging sites, whereas shy individuals should show greater variability in use of foraging sites, as they adapt to changing environmental conditions. Alternatively, boldness can lead to spatial partitioning, whereby individuals use mutually exclusive foraging areas (Patrick & Weimerskirch, 2014; Spiegel et al., 2015). If these foraging areas differ in resource predictability, different levels of site fidelity may emerge between bold and shy individuals as an artefact of spatial partitioning. Separating environmental and individual drivers of this relationship is important for elucidating the mechanisms linking personality to specialization.

In this study, we test whether boldness predicts individual differences in the degree of foraging specialization in black‐legged kittiwakes (*Rissa tridactyla*) breeding at four colonies in Svalbard. Kittiwakes are surface‐feeding seabirds which breed in socially monogamous pairs and exhibit biparental care, with both parents incubating eggs and provisioning for chicks until fledging at around 40 days (Coulson, 2011). Kittiwakes are known to show high inter‐individual differences in their foraging behaviour and to exhibit varying levels of foraging site fidelity (Irons, 1998; Suryan, Irons, & Benson, 2000). We first conducted standardized and repeated novel object tests to assess individuals' positions on the bold–shy continuum. Using GPS loggers, we then tracked the foraging movements of kittiwakes over a series of sequential trips to examine individual site fidelity. Specifically, we compared site fidelity in terms of consistent use of foraging locations at sea, and repeatability in the distance, duration and range of foraging trips. We then tested whether boldness leads to spatial partitioning of foraging sites, to examine whether differences in site fidelity are driven by spatial partitioning. If boldness predicts site fidelity but not spatial partitioning, this would indicate their linkage by individual, rather than environmental mechanisms.

## 2 MATERIALS AND METHODS

### 2.1 Study system

In 2017, we studied kittiwakes breeding at four colonies on the west coast of Svalbard: Blomstrand (78°59′N 12°07′E), Krykkjefjellet (78°53′N 12°11′E) and Observasjonholmen (78°56′N 12°16′E) in Kongsfjorden, and Grumant (78°10′N 15°05′E) in Isfjorden. Kittiwakes build cup‐shaped nests from mud and vegetation (Coulson, 2011). At Grumant, kittiwakes nest on the window ledges of an abandoned building, while at the Kongsfjorden colonies, kittiwakes nest on the ledges of natural cliffs (see Appendix S1 for more details). Molecular sexing was conducted on DNA extracted from blood and feather samples (Appendix S2). All but two individuals were first caught as breeding adults, and birds were therefore of unknown age.

### 2.2 Boldness tests

We measured individual boldness in response to a novel object, a method routinely used to assess boldness in colonial seabirds (Grace & Anderson, 2014; Patrick & Weimerskirch, 2014) including black‐legged kittiwakes (Collins, Hatch, Elliott, & Jacobs, 2019). A full‐field protocol is provided in Appendix S3. An observer presented a novel object (a blue plastic penguin toy, dimensions 13 × 10 × 4.5 cm; Munchkin®) to birds on their nests. The object was mounted on the end of an 8‐m carbon fibre fishing pole, with an action camera (GoXtreme® WiFi) fixed 30 cm behind the object recording birds' responses. The observer held the opposite end of the pole from the ground level. Before beginning the test, the observer positioned the novel object at ground level directly beneath the position of the focal nest, where it was out of view of the colony. The observer then raised the object at a constant pace directly upwards towards the nest, until the object rested on the cup of the nest, over a period of 30 s. The object was held in position for 60 s, before retracting the object and returning it to ground level. Tests were conducted during incubation and early chick rearing. Tests were conducted only when a single adult was attending the nest. Repeat tests were conducted whenever possible after a minimum of 2 days, subject to the presence of the focal individual on the nest. A single observer conducted all tests in Isfjorden and a second in Kongsfjorden. Videos were analysed blindly by a single observer using jwatcher v1.0 (Blumstein & Daniel, 2007). From the second the object reached the height of the nest, we recorded the proportion of the subsequent 60 s the focal bird spent in each of five mutually exclusive behavioural states: (a) sitting on the nest, with the body resting on the nest cup; (b) body raised off nest cup, but not standing; (c) standing on the nest (legs visible and extending to the base of the nest); (d) off the nest but remaining on the cliff or window ledge close to the nest; and (e) off the cliff or window ledge (and no longer visible). A total of 133 individuals were tested: 80 were tested once, 29 were tested twice, 15 were tested three times, and 9 were tested more than 3 times (totalling 53 individuals tested more than once).

### 2.3 GPS tracking

We used GPS loggers to track 50 kittiwakes during incubation and 54 kittiwakes during chick rearing, 19 of which were tracked in both breeding stages. All but one tracked individuals were personality tested (Appendix S1 Table S1). Loggers were programmed to record a location every 10 min on incubating birds and every 2 min on chick‐rearing birds (this was to ensure sufficient battery life to record multiple trips per bird during incubation, as incubation trips were known to be substantially longer than chick‐rearing trips) (mean duration 15 hr vs. 5 hr; see also Robertson, Bolton, Grecian, & Monaghan, 2014). At one colony (Krykkjefjellet), loggers were also programmed to a 10‐min resolution during chick rearing to meet the data requirements of another study. Birds were equipped with one of three logger types (i‐gotU GT‐120, Mobile Action©; CatLog Gen1 and CatLog Gen2, both http://www.mr-lee.com/sc_supp.htm), a subset of which were refitted with a smaller battery to reduce mass (Appendix S4 Table S4). Loggers were sealed in waterproof heat shrink tubing and attached to birds' back feathers using TESA tape and including attachment materials ranged from 6.3 to 18.6 g in mass (1.5%–4.6% of a kittiwake's body mass). We tested whether differences in logger mass influenced foraging behaviour by modelling its effect on the distance, duration and range of foraging trips. We detected no relationship between logger mass and foraging behaviour (Appendix S4), and therefore do not discuss these results further in the main results.

Owing to distinctly different foraging behaviour between incubation and chick‐rearing periods (Robertson et al., 2014; Table 1), data were analysed separately by breeding stage. During data processing, we removed points within a 300 m buffer of each colony (based on the frequency histogram of point distance to the colony) and defined foraging trips as periods longer than 1 hr spent outside this buffer (based on the frequency histogram of trip durations; Warwick‐Evans et al., 2016). Trips longer than 1 hr may still include trips carried out for purposes besides foraging, such as bathing. To restrict analyses to foraging trips only, we visually inspected all trips for evidence of detectable foraging behaviour. Seabirds use area‐restricted search (ARS) to locate prey, during which movements are characterized by reduced speeds and increased tortuosity (Fauchald & Tveraa, 2003). A small number of trips (*N* = 10; 4 by bold individuals and 6 by shy individuals) contained no evidence of ARS and were consequently removed from all analyses. All 10 trips were considerably shorter than the mean trip duration (1.5 hr vs. 10 hr), which supported that these movements were likely not foraging trips. In total, we recorded 111 foraging trips from 50 individuals during incubation, in 31 of which more than one trip was recorded for, and 212 foraging trips from 54 individuals during chick rearing, in 45 of which more than one trip was recorded for. All individuals with multiple trips recorded were personality tested (one individual with a single trip recorded during incubation was not personality tested). To standardize data resolution and to account for occasional missing GPS points, we used adehabitatLT (Calenge, 2015) to linearly interpolate tracks to intervals of 10 min during incubation and 2 min during chick rearing.

**Table 1:** Summary of foraging statistics (mean ± *SE*) for each colony during incubation and chick rearing

Colony	Trip metric	Incubation	Chick rearing
Grumant	Distance (km)	552.70 ± 87.12	196.32 ± 23.53
	Duration (hr)	29.52 ± 4.25	9.78 ± 1.04
	Maximum range (km)	186.66 ± 28.92	75.70 ± 8.49
Blomstrand	Distance (km)	147.44 ± 70.53	47.12 ± 7.76
	Duration (hr)	16.84 ± 3.85	4.28 ± 0.41
	Maximum range (km)	55.70 ± 28.99	15.62 ± 2.32
Krykkjefjellet	Distance (km)	43.02 ± 9.79	25.10 ± 2.06
	Duration (hr)	11.80 ± 2.88	4.48 ± 0.29
	Maximum range (km)	31.25 ± 9.57	8.73 ± 0.48
Observasjonholmen	Distance (km)	114.14 ± 37.66	31.99 ± 2.16
	Duration (hr)	38.74 ± 14.78	3.72 ± 0.25
	Maximum range (km)	31.25 ± 9.57	8.43 ± 0.42

To identify foraging sites from GPS tracks, we classified each GPS point as one of three behavioural states using hidden Markov models (HMMs). Hidden Markov models are a type of state‐space model, which decompose observed time‐series data (here, movement) into an observed sequence of discrete behavioural states. Hidden Markov models were fitted using the moveHMM function from the movehmm package (Michelot, Langrock, & Patterson, 2016), which we provided with starting parameters informed by previous work using HMMs to describe kittiwake foraging behaviour (Trevail et al., 2019; Appendix S5). Based on the distributions of step lengths between GPS points (described by a gamma distribution) and turning angles (described by a von Mises distribution) between consecutive GPS points, HMMs classified each point as one of three behavioural states: foraging, resting or travelling. We used the Viterbi algorithm to estimate the most likely sequence of states to have generated the observed movement patterns (Zucchini, MacDonald, & Langrock, 2016). A three‐state model was supported by model selection using AIC, and the three states and their interpretation are consistent with other kittiwake‐tracking studies (Chivers et al., 2012; Trevail et al., 2019). Consecutive sequences of foraging points were aggregated into foraging sites and were represented by a single pair of central coordinates (Appendix S5). In total, we identified 661 sites during incubation and 1,138 sites during chick rearing. Data were separated by breeding stage due to differential temporal data resolution and by fjord because distributions of step lengths and turning angles differed between the two fjords (Appendix S5), resulting in four HMMs in total.

### 2.4 Data analysis

We carried out analyses in r v3.5.1 (R Core Team, 2018), using the package lme4 (Bates, Mächler, Bolker, & Walker, 2014) for linear mixed‐effects models (LMMs). To determine statistical significance of fixed effects, we used ANOVA comparisons of models with and without each variable in turn. We checked model assumptions of normality and homoscedasticity by visual inspection of residual plots.

To estimate individual boldness, we used a principal component analysis (PCA) to collapse the five behaviour variables into a single test score (PC1). We estimated adjusted repeatability (Nakagawa & Schielzeth, 2010) of PC1 using the r package rptr (Stoffel, Nakagawa, & Schielzeth, 2017), including fixed effects to adjust for test date, breeding stage (incubation or chick rearing), observer and test number. To obtain a single estimate of boldness per individual, we extracted parameter estimates for each individual from a linear model. PC1 was fitted as the response variable, and individual ID, test date, breeding stage, observer and test number were fitted as fixed effects. We tested for sex differences in boldness estimates in a linear model with sex as a fixed effect.

To quantify foraging site fidelity, we calculated a similarity index following Patrick and Weimerskirch (2017). Briefly, with each site used in turn as the focal site, we randomly paired the focal site with (a) one site used by the same individual on a different foraging trip (within‐individual paired site) and (b) one site from each other individual from the same colony (between‐individual paired sites). Site fidelity was estimated only for individuals with more than one trip recorded (*N* = 31 during incubation; *N* = 45 during chick rearing), but single‐trip birds were retained as between‐individual pairs, to compare the focal individual with the full tracked population. The similarity index was then the proportion of between‐individual paired sites that were closer to the focal site than the within‐individual paired site (Appendix S5). The index is bounded between 0 and 1, and for interpretability, this was inverted (1 − *x*) so that values towards 1 indicate high site fidelity (no other individuals foraging more closely to the focal site than the individual's own paired site) and towards 0 indicate low site fidelity (all individuals foraged more closely to the focal site than the individual's own paired site). We ran 1,000 iterations of the randomization, such that each focal site was randomly paired 1,000 times. For each model iteration, we then fitted a binomial generalized linear model (GLM) with individual ID as a fixed effect to extract a single estimate and standard error of site fidelity for each individual across all of its foraging sites. The time difference (number of days) between paired sites was also included as a fixed effect, to account for variation in temporal proximity between pairs. This resulted in 1,000 estimates of site fidelity per individual. Finally, we examined the predictors of site fidelity using a linear model with the following structure: boldness, sex, colony and date were fitted as fixed effects, and the two‐way interactions between boldness and sex, and boldness and colony were included. Site fidelity was fitted as the response variable, and as the randomization generated 1,000 estimates of site fidelity per individual (one from each iteration of the randomization), the model was run 1,000 times also, using a loop to set the site fidelity estimates produced by each iteration of the randomization (Patrick & Weimerskirch, 2015). We present 95% confidence intervals for model estimation based on the 1,000 model iterations (Nicolaus et al., 2012).

To test for spatial partitioning by boldness, we examined whether boldness predicted the latitudes and longitudes of foraging sites, to test whether variation in boldness was associated with geographic variation in foraging sites. To do this, we modelled the central latitude (log2 transformed to approach normality) and longitude (square‐root transformed to approach normality) of foraging sites using LMMs. Trip ID nested within bird ID was fitted as a random effect to adjust for multiple foraging sites within a trip and multiple trips per individual. Boldness, sex, colony and date were fitted as fixed effects, and the two‐way interactions between boldness and sex, and boldness and colony were included. We additionally tested for a relationship between boldness and the extent to which a bird's foraging distribution overlapped with the colony‐level distribution and found no evidence for a relationship (see Appendix S6).

Wide‐ranging animals such as seabirds can be specialized in aspects of space use besides spatial locations of foraging behaviour. To quantify other measures of foraging specialization, we examined three summary metrics of foraging trips: (a) mean foraging trip distance (km); (b) mean foraging trip duration (hours); and (c) mean maximum range from the colony (km). Occasionally recordings of foraging trips were incomplete due to logger failure (*N* = 39). These trips were excluded from our calculations of trip distance and duration, and were only included in calculation of maximum range from the colony if the bird had returned within 75% of the maximum distance from the colony before logger failure (*N* = 18; Paredes et al., 2012). To test whether boldness was associated with specialization in each foraging trip metric, we grouped individuals by boldness scores and compared repeatability of trip metrics between groups, since repeatability is a group‐level measure of individual consistency (Nakagawa & Schielzeth, 2010). Based on the median boldness score, birds were categorized as either ‘bold’ (higher values) or ‘shy’ (lower values), resulting in 67 bold individuals and 66 shy individuals. We estimated repeatability of trip distance, duration and maximum range (all log10 transformed) for bold and shy birds separately, and checked for non‐overlapping 84% confidence intervals between bold and shy birds, since the absence of overlap between 84% confidence intervals is equivalent to a *z* test at the 0.05 level (Aplin et al., 2015; Payton, Greenstone, & Schenker, 2003; Tryon, 2001).

To avoid issues pertaining from multicollinearity, we were unable to include both date and chick age as fixed effects in models on chick‐rearing data. While birds may adjust foraging behaviour with chick age (Christensen‐Dalsgaard et al., 2018), bold and shy kittiwakes did not differ in the age of their chicks at logger deployment (Appendix S4), and therefore, any detected effects of boldness are unlikely to be mediated by differences in chick age at tracking.

## 3 RESULTS

### 3.1 Boldness

PC1 explained 61% of the variance in the response to the novel object (see Table 2 for variable loadings). Boldness scores ranged from −1.690 to 1.519 with low values representing instances when birds left the nest, medium values representing instances when birds remained on the nest but stood or raised up, and high values representing instances when birds did not adjust stance. Low values of PC1 were interpreted as ‘shy’ responses and high values as ‘bold’ responses. Kittiwakes were highly repeatable in response to the novel object (*R* = .678, CI: 0.572–0.791; *p* < .001). We detected no difference in boldness between the sexes (*F*_1,129_ = 2.863; *p* = .098).

**Table 2:** Principal component analysis output for boldness scores

Behaviour	PC1	PC2	PC3	PC4	PC5
Sitting	0.743	0.462	−0.118	0.144	0.447
Raised up	−0.001	−0.639	−0.600	0.178	0.447
Standing	−0.043	−0.291	0.754	0.381	0.447
Off the nest	−0.032	−0.069	0.151	−0.878	0.447
Off the ledge	−0.667	0.537	−0.186	0.175	0.447
Cumulative variance explained	0.612	0.845	0.948	1.000	1.000

### 3.2 Site fidelity

**Figure 1:**
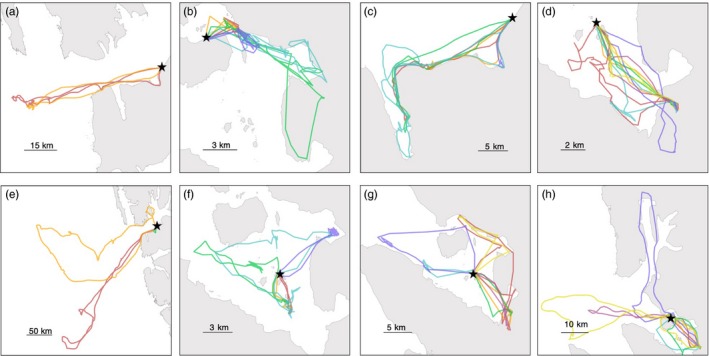
Differences in foraging specialisation between bold and shy kittiwakes. Top row shows repeated foraging trips from four different bold individuals (a: *N* = 2 trips; b: *N* = 5 trips; c: *N* = 5 trips; d: *N* = 7 trips). Bottom row shows repeated foraging trips from four different shy individuals (e: *N* = 3 trips; f: *N* = 5 trips; g: *N* = 7 trips; h: *N* = 7 trips). Trips are colour‐coded chronologically: 1 = red; 2 = orange; 3 = green; 4 = blue; 5 = purple; 6 = pink; 7 = yellow. Colony locations are marked by black stars

**Figure 2:**
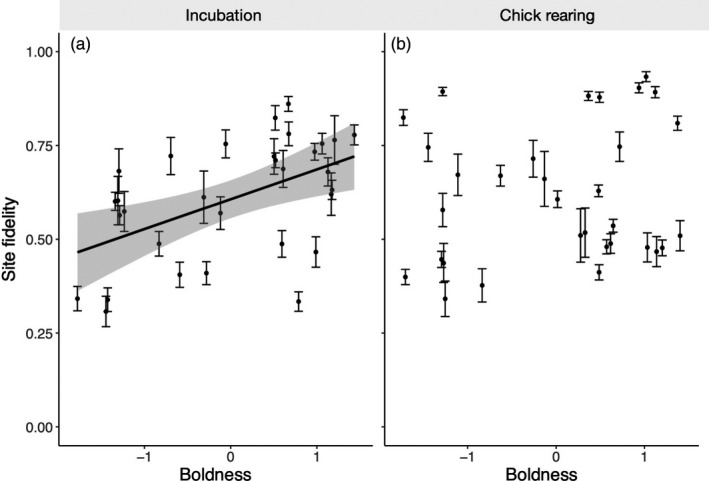
The relationship between boldness and foraging site fidelity. Data were separated by breeding stage into incubation foraging trips (a) and chick‐rearing foraging trips (b). High values indicate highly site‐faithful individuals. We present mean values of site fidelity (±*SE*) for each individual. Bolder individuals showed lower estimates of foraging site fidelity during incubation (a) but not during chick rearing (b)

**Figure 3:**
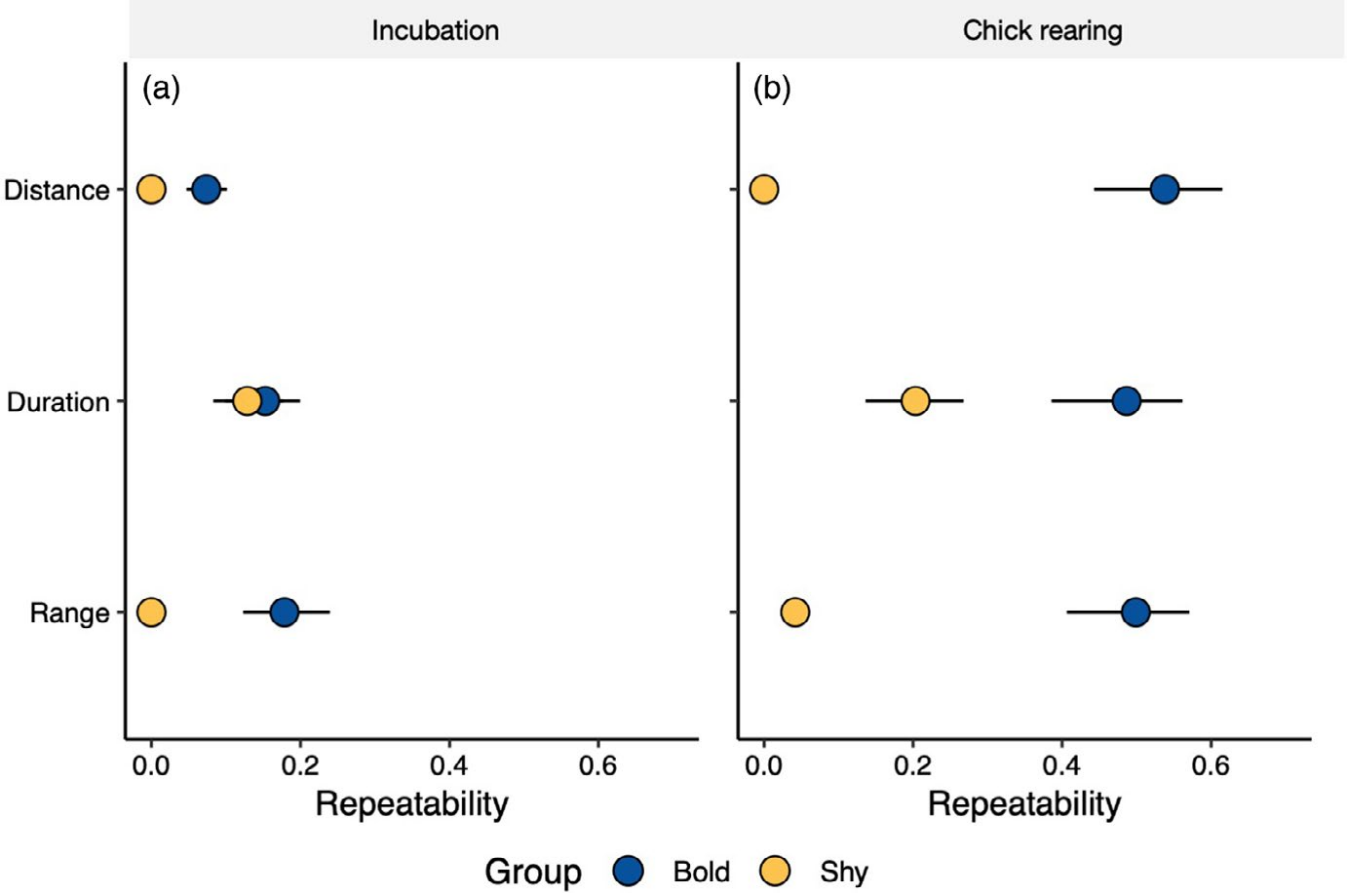
Repeatabilities of the distance, duration and maximum range of foraging trips made by shy and bold birds. Results are shown during incubation trips (a) and chick‐rearing trips (b). Dark blue points indicate bold individuals, and yellow points indicate shy individuals. While boldness is a continuous measure in other analyses, here individuals were grouped by boldness to be able to compare differences in repeatability (since repeatability is a group‐level measure). 84% confidence intervals are displayed: non‐overlapping 84% confidence intervals are equivalent to *z* tests at the 0.05 level

Site fidelity was variable between birds during both breeding stages (incubation: median = 0.588, range = 0.291–0.846; chick rearing: median = 0.554, range = 0.332–0.933; Figure 1). Boldness was positively related to site fidelity during incubation (*F*_1,25_ = 13.391, *p* = .003; Figures 2 and 3; Table 3), with bolder individuals showing higher faithfulness to foraging sites than shy individuals. No relationship between boldness and site fidelity was found during chick rearing (Figure 3; Table 3). There was no interaction between boldness and sex (incubation: *F*_1,21_ = 0.287, *p* = .689; chick rearing: *F*_1,32_ = 0.178, *p* = .739) or between boldness and colony (incubation: *F*_3,23_ = 0.912, *p* = .512; chick rearing: *F*_3,34_ = 0.692, *p* = .585) on site fidelity.

**Table 3:** Results for the effects of boldness, sex, date and colony on site fidelity and spatial partitioning (latitudinal and longitudinal locations of foraging sites)

	Response	Model output	Boldness	Sex (male)	Date	Colony
Incubation	Site fidelity	Estimate ± *SE*	0.086 ± 0.024	0.059 ± 0.064	0.036 ± 0.081	
		Test statistic	*F*_1,25_ = 13.391	*F*_1,25_ = 1.333	*F*_1,25_ = 1.812	*F*_3,27_ = 2.493
		*p* value	***p* = .003**	*p* = .359	*p* = .264	*p* = .130
		Estimate range	0.085–0.087	0.053–0.064	0.027–0.045	
	Site latitude	Estimate ± *SE*	−0.059 ± 0.036	−0.048 ± 0.076	0.039 ± 0.038	
		Test statistic	$\chi_{1}^{2}$ = 2.855	$\chi_{1}^{2}$ = 0.382	$\chi_{1}^{2}$ = 0.890	$\chi_{3}^{2}$ = 109.310
		*p* value	*p* = .097	*p* = .537	*p* = .346	***p* < .001**
	Site longitude	Estimate ± *SE*	−0.028 ± 0.041	−0.177 ± 0.086	−0.022 ± 0.044	
		Test statistic	$\chi_{1}^{2}$ = 0.477	$\chi_{1}^{2}$ = 4.398	$\chi_{1}^{2}$ = 0.307	$\chi_{3}^{2}$ = 5.694
		*p* value	*p* = .490	***p* = .036**	*p* = .580	*p* = .128
Chick rearing	Site fidelity	Estimate ± *SE*	0.005 ± 0.040	0.098 ± 0.076	−0.027 ± 0.185	
		Test statistic	*F*_1,36_ = 0.097	*F*_1,36_ = 1.768	*F*_1,36_ = 0.110	*F*_3,38_ = 0.782
		*p* value	*p* = .811	*p* = .232	*p* = .794	*p* = .544
		Estimate range	0.004–0.005	0.093–0.102	−0.038 to −0.015	
	Site latitude	Estimate ± *SE*	0.009 ± 0.006	−0.030 ± 0.012	0.009 ± 0.008	
		Test statistic	$\chi_{1}^{2}$ = 2.531	$\chi_{1}^{2}$ = 6.075	$\chi_{1}^{2}$ = 1.216	$\chi_{3}^{2}$ = 456.020
		*p* value	*p* = .112	***p* = .014**	*p* = .270	***p* < .001**
	Site longitude	Estimate ± *SE*	0.008 ± 0.017	−0.015 ± 0.035	0.047 ± 0.024	
		Test statistic	$\chi_{1}^{2}$ = 0.210	$\chi_{1}^{2}$ = 0.175	$\chi_{1}^{2}$ = 3.058	$\chi_{3}^{2}$ = 45.548
		*p* value	*p* = .647	*p* = .676	*p* = .054	***p* < .001**

Note

Significant terms are indicated in bold. Two‐way interactions between boldness and sex, and boldness and colony were found to be non‐significant and dropped from all models (results presented in the text). Estimates for sex effects are presented as the difference for males over females. Estimate range for site fidelity models is the 95% confidence intervals extracted from a model that uses 1,000 estimates of site fidelity per individual, included to incorporate individual variability in site fidelity.

### 3.3 Foraging trip repeatability

Foraging trips were longer in duration and further in distance and range during incubation compared to during chick rearing (Table 1). During incubation, bold kittiwakes were more repeatable than shy birds in foraging trip duration (bold: *R* = .162, CI = 0.113, 0.208; shy: *R* = .051, CI = 0.032, 0.085) and range (bold: *R* = .185, CI = 0.129, 0.243; shy: *R* = .001, CI = 0.000, 0.001; Figure 3a), while foraging trip distance was not repeatable regardless of personality (*R* = .072). During chick rearing, bold kittiwakes were more repeatable in foraging trip distance (bold: *R* = .543, CI = 0.466, 0.624; shy: *R* = .000, CI = 0.000, 0.000), duration (bold: *R* = .502, CI = 0.401, 0.587; shy: *R* = .130, CI = 0.098, 0.184) and maximum range (bold: *R* = .494, CI = 0.403, 0.575; shy: *R* = .029, CI = 0.011, 0.038; Figure 3b).

### 3.4 Boldness and spatial partitioning of foraging distributions

We found no evidence for spatial partitioning by boldness in kittiwakes, as boldness did not predict the latitude and longitude of foraging sites during either breeding stage. We found no evidence for interacting effects of boldness with sex on spatial partitioning (incubation: boldness × sex on latitude: $\chi_{1}^{2}$ = 0.121, *p* = .729; boldness × sex on longitude: $\chi_{1}^{2}$ = 1.276, *p* = .259. Chick rearing: boldness × sex on latitude: *F*_1,46_ = 0.257, *p* = .614; boldness × sex on longitude: $\chi_{1}^{2}$ = 3.156, *p* = .076), or boldness and colony (incubation: boldness × colony on latitude: $\chi_{3}^{2}$ = 6.127, *p* = .106; boldness × colony on longitude: $\chi_{3}^{2}$ = 2.214, *p* = .529. Chick rearing: boldness × colony on latitude: $\chi_{3}^{2}$ = 3.707, *p* = .295; boldness × colony on longitude: $\chi_{3}^{2}$ = 0.530, *p* = .912). Females utilized sites further east than males during incubation (Table 3).

## 4 DISCUSSION

Individual differences in foraging specialization were linked to boldness in black‐legged kittiwakes across multiple colonies. Individual kittiwakes varied in their level of foraging site fidelity, and in line with our predictions, bolder kittiwakes exhibited higher foraging site fidelity than shy individuals, providing the first demonstration that personality is related to site fidelity. This relationship was present during incubation but not chick rearing. In addition, during both incubation and chick rearing, bolder birds were more repeatable in their foraging trips than shy individuals, indicating that bold individuals were more specialized, and shy individuals more generalized, in their behaviour. We found no evidence of boldness‐dependent spatial partitioning: boldness was not associated with foraging at particular latitudes or longitudes, indicating that bold and shy individuals exhibited different levels of specialization while foraging over the same areas. Together, these results suggest that personality differences may constitute important predictors of differences in individual foraging specializations.

### 4.1 Differences between breeding stages

In keeping with previous work on kittiwakes (Irons, 1998), we observed individual differences in foraging site fidelity, demonstrating the coexistence of specialist and generalist foraging strategies. Median site fidelity did not differ between incubation and chick rearing, but we found that birds were markedly more repeatable in the distance, duration and range of their foraging trips during chick rearing compared to during incubation. Shifts in foraging strategies between incubation and chick‐rearing periods have previously been reported in kittiwakes (Robertson et al., 2014) and may result from seasonal changes in resource availability, for example due to the depletion of prey patches (Birt, Birt, Goulet, Cairns, & Montevecchi, 1987). However, we found no evidence of a linear change in site fidelity with date, which would indicate behavioural changes to match shifting resource distributions. Instead, we suggest that increased consistency during chick rearing is likely linked to concomitant reductions in trip length, due to the increased demands of the chick‐rearing period (Weimerskirch, Salamolard, Sarrazin, & Jouventin, 1993). During incubation in many seabird species, birds make longer trips to profitable foraging grounds that are presumably out of reach after hatching, when time spent away from the nest is constrained by offspring demand for provisioning (Phillips et al., 2004; Robertson et al., 2014). Despite foraging trips being less consistent in length during incubation, average levels of site fidelity were similar in incubation to during chick rearing, demonstrating that returning to previous foraging locations is a favoured strategy even when adults are less constrained in their foraging movements.

### 4.2 Site fidelity without spatial partitioning

Previously, studies have linked boldness to spatial aspects of foraging, including home range size (Boon, Reale, & Boutin, 2008) and search methods (Wesley et al., 2012), but evidence linking personality to foraging site fidelity has been lacking. Our finding that bold individuals were more site‐faithful than shy individuals during incubation was coupled with a lack of spatial partitioning. The significance of a lack of spatial partitioning is that the relationship between boldness and site fidelity appears not to be driven by differences in habitat availability, at least at the broad spatial scale: instead, it suggests a behavioural difference between individuals occupying the same environment. Behavioural differences in foraging movements between bold and shy individuals are also evident in the fact that bold individuals were more repeatable in foraging trip metrics, during both incubation and chick rearing. Below, we outline potential causes of our finding of a relationship between breeding stages.

### 4.3 Boldness and foraging site fidelity

Shy birds were less site‐faithful than bold birds, but only during incubation, potentially owing to constraints on behavioural flexibility during the chick‐rearing period. As bold and shy individuals appear to share habitat availability, their differences in site fidelity during incubation suggest different responses to the environment. In predictable environments, returning to previous foraging locations should be favoured; conversely, in unpredictable habitats the probability of a previous location being profitable again is low, and consequently, animals should show lower site fidelity and greater reliance on environmental cues to locate prey (Switzer, 1993; Weimerskirch, Le Corre, Jaquemet, & Marsac, 2005). The marine environment is characterized by both persistent oceanographic features (bathymetric structures and fronts) which generate predictable prey patches, as well as highly dynamic tidal and weather processes which result in spatiotemporally variable resource distributions (Cox et al., 2016; Scales et al., 2014). High and low reliance on environmental cues may represent alternative foraging tactics that can both be profitable within the same macro‐scale habitat (Carroll, Harcourt, Pitcher, Slip, & Jonsen, 2018). Our findings suggest that shy and bold kittiwakes may differ in their propensity to adopt these two tactics during incubation, with bold individuals showing lower sensitivity to environmental cues than shy individuals, but that during the chick‐rearing period, shy individuals switch to a high site fidelity foraging strategy. Shyer animals are often characterized by high responsiveness to change (Coppens et al., 2010; Wolf et al., 2008), and indeed, in our boldness test, shy individuals were more responsive to the presentation of a novel object. Previous work has linked boldness with responsiveness to environmental change: for instance, shy, but not bold, Atlantic cod (*Gadus morhua*) adjust their home ranges in response to increases in sea temperature (Villegas‐Ríos, Réale, Freitas, Moland, & Olsen, 2018), and in sleepy lizards (*Tiliqua rugosa*), shy individuals were more responsive to changes in resource availability (Spiegel et al., 2015). During incubation, when birds are less constrained to return to predictable foraging sites, shy individuals may therefore be more likely to select sites based on environmental cues, rather than based on previous foraging attempts. Reliance upon environmental cues may extend to social indicators of foraging opportunities, with some studies suggesting that shy individuals rely more heavily on social information when making foraging decisions (Aplin, Farine, Mann, & Sheldon, 2014; Kurvers et al., 2010).

Bolder, competitive individuals may make more use of reliable foraging patches (e.g. van Overveld et al., 2018). A study on black‐browed albatross (*Thalassarche melanophrys*) found that bold birds foraged in areas associated with high competition, while shy individuals avoided these regions (Patrick & Weimerskirch, 2014). Due to their increased propensity of bold animals to engage in competitive interactions (Dammhahn & Almeling, 2012; Sih, Bell, & Johnson, 2004), bold kittiwakes may consistently use predictable foraging hotspots, while shy individuals avoid such areas when able to do so and instead forage more variably in less predictable habitat. In Arctic waters, glacial zones constitute key foraging habitat for kittiwakes and represent highly predictable and detectable foraging areas (Lydersen et al., 2014). Accordingly, glaciers may represent such foraging hotspots that could be disproportionately used by bold and not shy kittiwakes in Svalbard. The next step to test for personality‐dependent habitat selection requires models of oceanographic conditions across the population's foraging range, to examine whether shy and bold kittiwakes select foraging areas associated with different levels of predictability and competition.

While shy animals typically exhibit flexibility in response to environmental fluctuations, bolder animals are instead thought to rely upon routines when navigating (Benus et al., 1990; Coppens et al., 2010; Marchetti & Drent, 2000). For example, bold great tits (*Parus major*) were found to quickly develop a routine‐like search pattern of feeding sites and were robust in following routines even when sites have been unprofitable on previous visits, while shy birds were more likely to visit new sites (Verbeek et al., 1994). When locating foraging sites, bold kittiwakes may navigate by routine‐like behavioural tendencies, resulting in higher return rate to previously visited sites. Our results comparing repeatability of bold and shy birds' foraging trips lend further support to this suggestion: bold kittiwakes made foraging trips that were more consistent in distance, duration and range than shy individuals, which may reflect routine‐like usage of the same routes to foraging sites in bolder birds. Interestingly, during chick rearing, bold birds continued to make foraging trips that were markedly more consistent in length than the trips of shy individuals, while bold and shy birds showed no differences in site fidelity. This suggests even when constrained by offspring provisioning to return to known reliable sites, bold and shy birds differ in how they navigate to these locations.

## 5 CONCLUSIONS

Our study found that a widely studied personality trait, boldness, predicts more specialized foraging behaviour during incubation in four colonies of kittiwakes. Studies of specialized foraging behaviour often overlook variation in individuals' level of specialization (but see Grecian, Lane, Michelot, Wade, & Hamer, 2018; Patrick & Weimerskirch, 2017; Votier et al., 2017; Wakefield et al., 2015). While site fidelity may have consequences for individual fitness (Authier, Bentaleb, Ponchon, Martin, & Guinet, 2012; Patrick & Weimerskirch, 2017), coexistence of specialists and generalists suggests that site fidelity may be under fluctuating selection (van de Pol et al., 2010; Wilson & Yoshimura, 1994) or frequency‐dependent selection (Fitzpatrick, Feder, Rowe, & Sokolowski, 2007). Here, we suggest that individual differences in site fidelity may also be maintained through association with personality traits. In another seabird species, boldness has been shown to be a heritable trait repeatable between years (Patrick, Charmantier, & Weimerskirch, 2013) and therefore has the potential to result in differences in foraging behaviour under selection. Despite an increased move towards individual‐based approaches in foraging and movement ecology, individual drivers of variation in behaviours such as site fidelity are commonly overlooked, and the number considering factors beyond age and sex is even rarer. Future studies on individual foraging specializations should also consider examining the combined effects of personality differences with other factors, such as age, or variability in environmental factors such as prey distribution. We advocate that consideration of phenotypic‐level behavioural differences such as differences in boldness is important in improving understanding of variation in behavioural specialization.

## AUTHORS' CONTRIBUTIONS

S.M.H., S.C.P., S.D. and L.U.S. conceived the study and the methodology. S.M.H. conducted statistical analysis and wrote the manuscript. S.M.H, S.C.P., S.D., P.B. and O.C. conducted the fieldwork. All authors contributed substantially to production of the manuscript.

## Data Availability

Data are accessible from the Dryad Digital Repository: https://doi.org/10.5061/dryad.221f9g2 (Harris et al., 2019).
