# Supplementary material for: Personality predicts foraging site fidelity and trip repeatability in a marine predator
Source: J Anim Ecol. 2019 Oct 18;89(1):68–79. doi: 10.1111/1365-2656.13106 (PMC7004082; doi:10.1111/1365-2656.13106)
Supplement: Supplementary file 1 [file JANE-89-68-s001.docx]

**Personality predicts foraging site fidelity and trip repeatability in a marine predator**

Stephanie M. Harris, Sébastien Descamps, Lynne U. Sneddon, Philip Bertrand, Samantha C. Patrick

Supporting Information

[**Appendix S1: Colony details** II](#_Toc20732639)

[**Appendix S2: Molecular sexing methodology** III](#_Toc20732640)

[**Appendix S3: Boldness test protocol** IV](#_Toc20732641)

[**Appendix S4: GPS tracking** V](#_Toc20732642)

[*S4.1* *GPS logger models and masses* V](#_Toc20732643)

[*S4.2* *Effects of logger mass on foraging behaviour* V](#_Toc20732644)

[*S4.3* *Variation in chick age at logger deployment* V](#_Toc20732645)

[**Appendix S5: Hidden Markov models and foraging site fidelity** VI](#_Toc20732646)

[**Appendix S6: Testing for an effect of boldness on foraging distribution overlap** IX](#_Toc20732647)

[**References** X](#_Toc20732648)

# **Appendix S1: Colony details**

**Table S1.** Details of black-legged kittiwake *Rissa tridactyla* colonies studied in 2017. Colony size is the estimated number of breeding pairs in 2017. Number of kittiwakes tested for boldness, tracked during incubation, and tracked during chick rearing are presented by colony, along with total across all colonies, and total across all colonies that were tested more than once/tracked for more than one trip at the bottom. ^1^Numbers in parentheses indicate number of tracked individuals that were also tested for boldness. Not all personality tested individuals were tracked, and not all tracked individuals were personality tested, but all personality tested individuals (including those not tracked) were included in the estimation of boldness and its repeatability; all tracked individuals (including those not boldness tested) were included the estimation of individual foraging site fidelity.

| **Fjord**  **Colony** | **Coordinates** | | **Colony size** | **N. boldness tested** | **N. tracked: incubation** | **N. tracked: chick rearing** |
| --- | --- | --- | --- | --- | --- | --- |
| Isfjorden |  | |  |  |  |  |
| Grumant | 78°10’N 15°05’E | | 50 | 62 | 16 (16) | 27 (27) |
| Kongsfjorden |  | |  |  |  |  |
| Blomstrand | 78°59’N 12°07’E | | 900 | 25 | 9 (9) | 5 (5) |
| Krykkjefjellet | 78°53’N 12°11’E | | 200 | 19 | 8 (7) | 9 (8) |
| Observasjonholmen | 78°56’N 12°16’E | | 150 | 27 | 17 (17) | 13 (13) |
| **Total** | |  |  | **133** | **50 (49)** | **54 (54)** |
| **Total w/ repeat measures** | |  |  | **53** | **31 (31)** | **45 (45)** |

# **Appendix S2: Molecular sexing methodology**

Sex was determined after DNA extraction and polymerase chain reaction (PCR). Genomic DNA was extracted from blood and feathers using DNeasy 96 Blood and Tissue Kit (Qiagen, Hilden, Germany) following the manufacturer’s protocol. Sex was determined using the primers M5 (Bantock, Prys-Jones, & Lee, 2008) and P8 (Griffiths, Double, Orr, & Dawson, 1998). These primers amplify the sex-linked CHD-W and CHD-Z genes, which differ in length and result in a single band for males and two bands for females. The M5 primer was 6FAM fluoro-labelled. Polymerase chain reaction (PCR) was performed with Qiagen’s Multiplex PCR Kit following the manufacturer’s protocol, but using 8.4 µL reaction volume. PCR products were mixed with GeneScan 500 LIZ (Applied Biosystems) size standard and Hi-Di formamide. Alleles were separated using capillary electrophoresis on an ABI 3500xl Genetic Analyzer and sizes assigned using GeneMapper software (Applied Biosystems).

# **Appendix S3: Boldness test protocol**

Protocol for measurement of boldness in the field:

- Fully extend the pole before beginning the test. Ensure no other person is in front of the colony before beginning.
- When ready to test a bird, start the video recording and note down the exact time on the camera.
- Position yourself in front of the position of the focal bird’s nest, with the object at ground level beneath the nest.
- During the test, minimise all movements besides raising the pole, refrain from speaking, and avoid staring directly at birds.
- Slowly and steadily, over approx. 30s, raise the pole towards the focal bird on the nest, taking care not to avoid sudden movements with the object or contact with the cliff/building. Aim to keep the pole at a 90° angle to the building as you raise it. Raise the pole by sliding it through the hands, wearing gloves if necessary to make the motion smooth.
- Bring the object to rest on top of the cup of the nest. Immediately begin timing and hold the object in place here for 60s.
- If the bird leaves the nest during the test, keep the object in place for the full 60s to record whether the bird returns.
- After 60s, carefully bring the object directly down to the ground, and stop the video recording. Move away from the colony before speaking.
- If two members of the pair are at the nest (i.e. during a changeover event), do not test either bird, but instead return later to test when only one bird is present.

Example videos of test responses can be found here:

<https://drive.google.com/drive/folders/13dTn4RaMo0F776PE4tQZ71soAR9RrwPN?usp=sharing>

# **Appendix S4: GPS tracking**

## *S4.1 GPS logger models and masses*

Kittiwakes were equipped with one of three different GPS logger models (i-GotU GT-120; CatLog Gen1, CatLog Gen2). To reduce logger mass, we modified a number of i-GotU and CatLog Gen1 loggers to carry a smaller battery (2.22 – 4.47g lighter). Sample sizes for each type of logger are provided in Table S4 (number deployed).

**Table S4**: Sample sizes of different GPS logger models and battery combinations

| **GPS logger model** | **Battery** | **Average mass (g) ± SD** | **Number used** |
| --- | --- | --- | --- |
| i-GotU GT-120 | Original | 17.75 ± 0.59 | 23 |
| i-GotU GT-120 | Modified | 13.28 ± 0.63 | 102 |
| CatLog Gen1 | Original | 15.10 ± 0.34 | 6 |
| CatLog Gen1 | Modified | 12.88 ± 0.43 | 10 |
| CatLog Gen2 | Original | 7.18 ± 0.44 | 33 |

## *S4.2 Effects of logger mass on foraging behaviour*

We tested whether differences in logger mass influenced foraging behaviour (foraging trip distance, duration, and maximum range from the colony), using linear models with the same model structure used in models of site fidelity and site selection. Distance, duration and range were all log10 transformed. Models were split by breeding stage. We found no effect of GPS logger mass on the distance (incubation: F_(1,37)_ = 0.84, p = 0.77; chick rearing: F_(1,43)_ = 0.13, p = 0.72), duration (incubation: F_(1,37)_ = 1.90, p = 0.17; chick rearing: F_(1,43)_ = 0.12, p = 0.73), and range of trips (incubation: F_(1,45)_ = 0.94, p = 0.34; chick rearing: F_(1,43)_ = 0.01, p = 0.91). This indicates variation in logger mass within the range used in our study was unlikely to drive differences in foraging behaviour.

## *S4.3 Variation in chick age at logger deployment*

The age of chicks at logger deployment (during the chick rearing phase) was variable (2-21 days), but we were unable to test of an effect of chick age due to strong collinearity with date. However, chick age at logger deployment did not correlate with boldness (F_(1,46)_ = 0.19, p = 0.66), hence effects of differences in chick age are unlikely to have biased our conclusions.

# **Appendix S5: Hidden Markov models and foraging site fidelity**

Owing to different resolutions of tracking data during incubation and chick rearing stages (10 min and 2 min intervals, respectively), we separated hidden Markov models (HMMs) by breeding stage. We additionally separated data by fjord (Isfjorden and Kongsfjorden), because kittiwake movement differed substantially in the distribution of step lengths and turning angles between the two fjords (see histograms in Figure S5.1 for incubation data and S5.2 for chick rearing data), and combined models resulted in poor classification of data. As a result, we ran four HMMs, the starting parameters for which are detailed in Table S5. Figures S5.1 and S5.2 show the HMM fitted behavioural state distributions classified overlaying the histograms of observed step length and turning angle distributions.


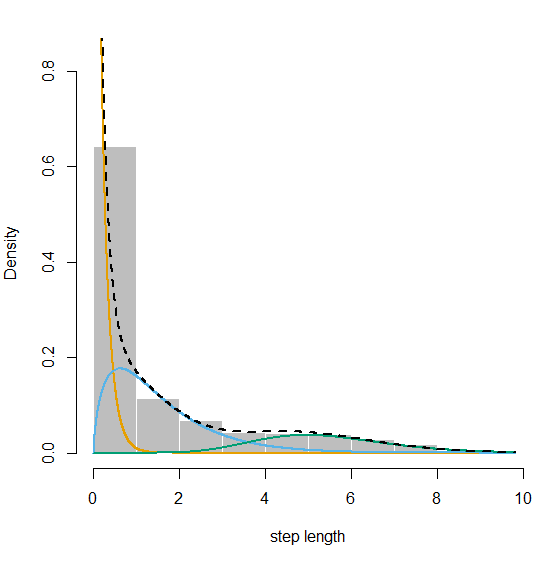

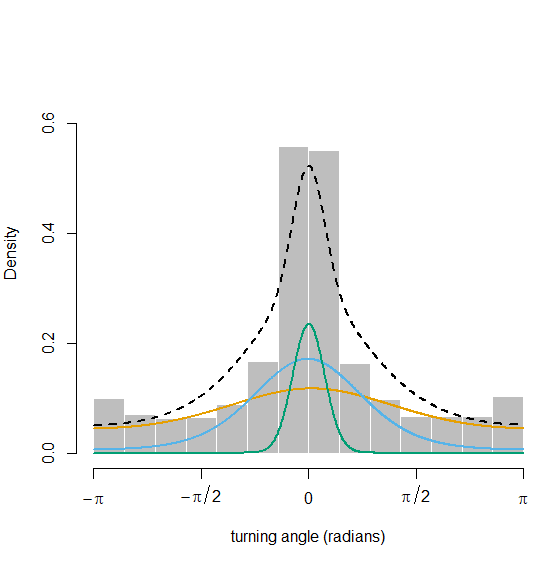


**–––** Resting

**–––** Foraging

**–––** Travelling


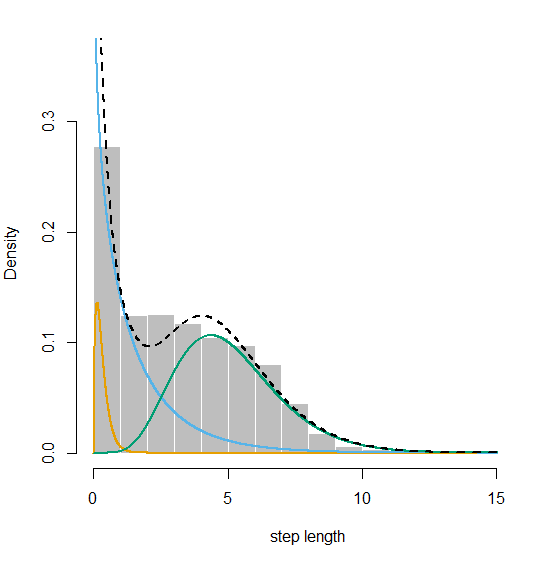

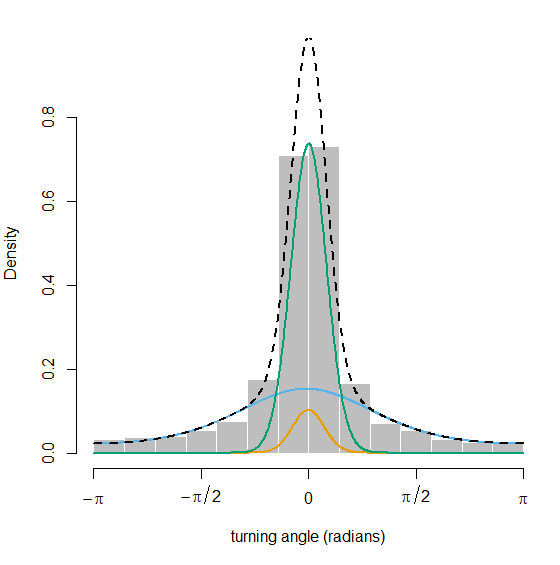


**Figure S5.1**: Histograms of (a, c) observed step lengths and (b, d) observed turning angles between consecutive GPS points during incubation for kittiwakes in Isfjorden (a, b) and Kongsfjorden (c, d). Coloured lines indicate HMM fitted state distributions (yellow: resting; blue: foraging; green: travelling).

(a)

(c)

(d)

(b)


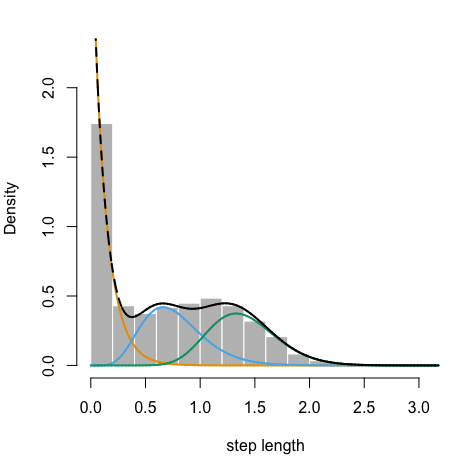

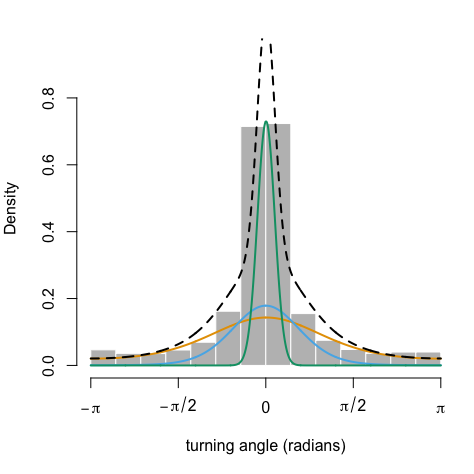

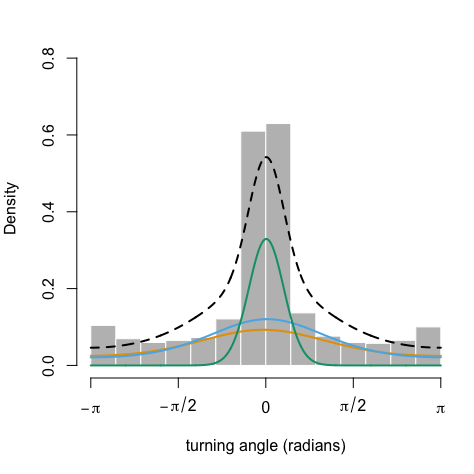

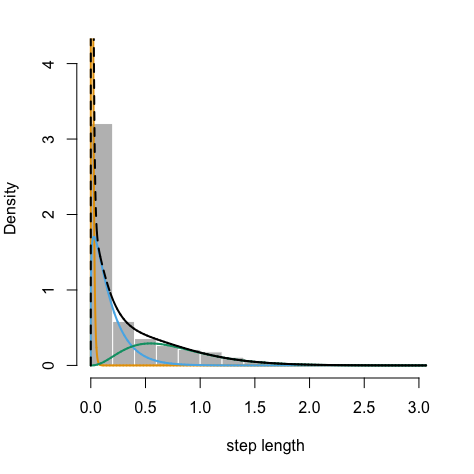


**–––** Resting

**–––** Foraging

**–––** Travelling

**Figure S5.2**: Histograms of (a, c) observed step lengths and (b, d) observed turning angles between consecutive GPS points during chick rearing for kittiwakes in Isfjorden (a, b) and Kongsfjorden (c, d). Coloured lines indicate HMM fitted state distributions (yellow: resting; blue: foraging; green: travelling).

(a)

(c)

(d)

(b)

**Table S5**. Starting parameters (SL: step length; TA: turning angle) for each HMM.

|  | **Incubation** | | **Chick rearing** | |
| --- | --- | --- | --- | --- |
| **Behaviour** | **Isfjorden** | **Kongsfjorden** | **Isfjorden** | **Kongsfjorden** |
| Resting | SL: 0.10 ± 0.20  TA: µ = 0, κ = 14 | SL: 0.20 ± 0.20km  TA: µ = 0, κ = 0.5 | SL: 0.02 ± 0.04  TA: µ = pi, κ = 0.5 | SL: 0.02 ± 0.04  TA: µ = pi, κ = 0.5 |
| Foraging | SL: 2.00 ± 1.50  TA: µ = 0, κ = 0.4 | SL: 2.00 ± 2.00km  TA: µ = pi, κ = 3.0 | SL: 0.15 ± 0.2  TA: µ = 0, κ = 0.5 | SL: 0.15 ± 0.2  TA: µ = 0, κ = 0.5 |
| Commuting | SL: 5.00 ± 2.00  TA: µ = 0, κ = 7 | SL: 5.00 ± 5.00  TA: µ = 0, κ = 0.5 | SL: 1.2 ± 0.5  TA: µ = 0, κ = 3 | SL: 1.2 ± 0.5  TA: µ = 0, κ = 3 |


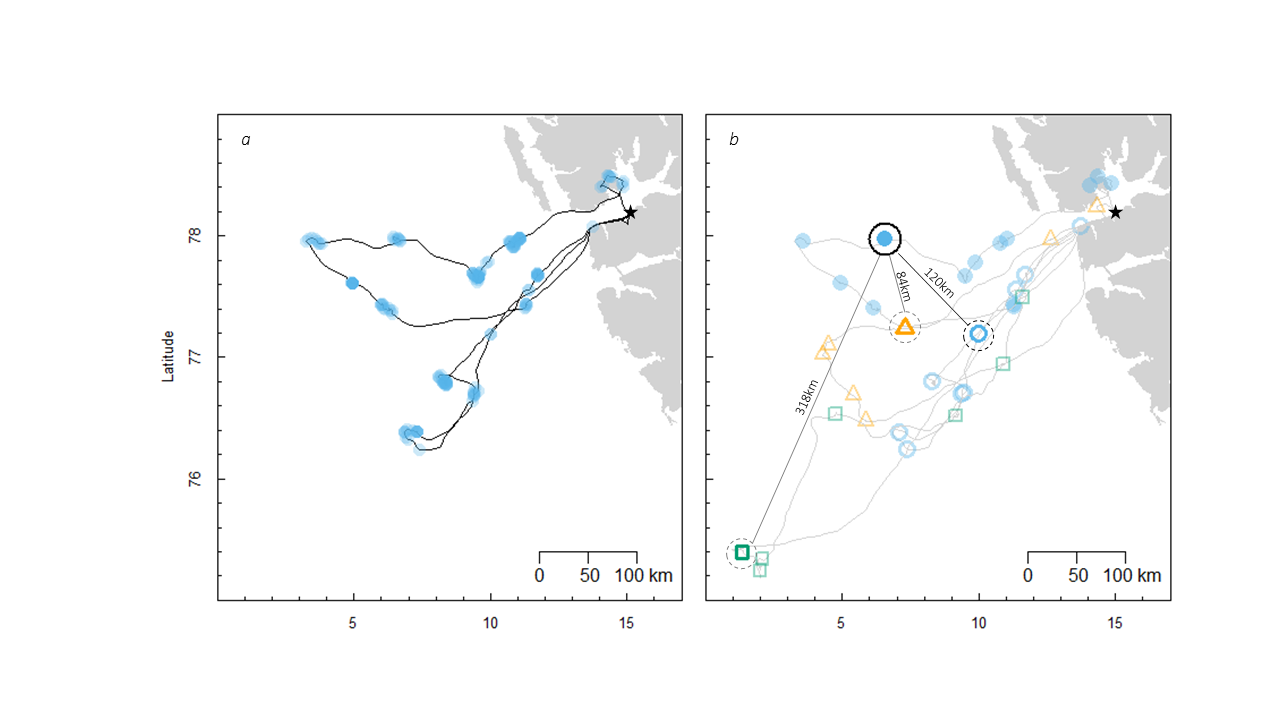


**Figure S5.3.** Methods used to estimate individual foraging site fidelity. a) First, GPS points on foraging trips (here two trips by the same individual are shown) were classified as either resting, travelling, or foraging behaviour (coloured in blue) using hidden Markov models. b) Consecutive sequences of points classified as foraging were deemed foraging sites, represented by the central coordinates of those sites. Here sites used by three birds are indicated by symbols (individual ID represented by symbol; open and filled symbols represent two different trips made by the same individual). For each site in turn as the focal site (here circled in black), focal sites were randomly paired with i) a within-individual site (a site used by the same individual on a different trip), and ii) a number of between individual sites (one site used by each other tracked individual). Here three paired sites are circled with a dashed line. The similarity index calculates the proportion of between-individual sites closer to the focal site than the within-individual site, here = 1/2 and so similarity = 0.5.

# **Appendix S6: Testing for an effect of boldness on foraging distribution overlap**

In addition to testing whether boldness was associated with linear geographic spatial partitioning, we used kernel density estimation (KDE) to test for a relationship between boldness and the extent to which bird’s foraging distributions overlap with that of the colony. We calculated 95% KDEs for each individual and the joint 95% KDE for all individuals within the same colony, with a grid size of approximately 5km^2^ and using the *href* algorithm to optimise the smoothing parameter (Calenge, 2006). Each individual’s overlap with the colony-level KDE was then estimated using the kerneloverlapHR function (Calenge, 2006), where 0 indicates no overlap and 1 indicates complete overlap with the colony -level foraging distribution. We tested whether KDE overlap was related to boldness by fitting it as the response variable in a linear model with boldness, sex, colony, and date were fitted as fixed effects, and the two-way interactions between boldness and sex, and boldness and colony included.

We found no evidence that boldness predicts individuals’ KDE overlap with the colony-level KDE (Table S6), nor an interaction between boldness and sex (incubation: F_1,42_ = 0.890, p = 0.351; chick rearing: F_1,46_ = 1.449, p = 0.235) or boldness and date (incubation: F_3,41_ = 0.471, p = 0.705; chick rearing: F_3,45_ = 0.331, p = 0.803) on KDE overlap.

**Table S6.2.** Results for the effects of boldness, sex, date, and colony on the overlap with colony-level kernel density estimation (95% KDE). Significant terms are indicated in bold. Two-way interactions between boldness and sex, and boldness and colony, were found to be non-significant and dropped from all models (results presented in the text). Estimates for sex effects are presented as the difference for males over females.

|  | **Incubation** | | | **Chick rearing** | | |
| --- | --- | --- | --- | --- | --- | --- |
| **Predictor** | **Estimate ± SE** | **Test statistic** | **P value** | **Estimate ± SE** | **Test statistic** | **P value** |
| Boldness | -0.02 ± 0.04 | F_1,43_ = 1.12 | p = 0.73 | -0.004 ± 0.04 | F_1,47_ = 0.02 | p = 0.90 |
| Sex (male) | 0.02 ± 0.04 | F_1,43_ = 1.12 | p = 0.73 | 0.013 ± 0.07 | F_1,47_ = 0.05 | p = 0.83 |
| Date | 0.02 ± 0.04 | F_1,43_ = 0.29 | p = 0.60 | -0.056 ± 0.05 | F_1,47_ = 1.50 | p = 0.23 |
| Colony |  | F_3,45_ = 9.77 | **p < 0.001** |  | F_3,49_ = 3.67 | **p = 0.02** |

# **References**

Bantock, T. M., Prys-Jones, R. P., & Lee, P. L. M. (2008). New and improved molecular sexing methods for museum bird specimens. *Molecular Ecology Resources*, *8*(3), 519–528. doi: 10.1111/j.1471-8286.2007.01999.x

Calenge, C. (2006). The package “adehabitat” for the R software: A tool for the analysis of space and habitat use by animals. *Ecological Modelling*, *197*(3–4), 516–519. doi: 10.1016/j.ecolmodel.2006.03.017

Griffiths, R., Double, M. C., Orr, K., & Dawson, R. J. G. (1998). A DNA test to sex most birds. *Molecular Ecology*, *7*(8), 1071–1075. doi: 10.1046/j.1365-294x.1998.00389.x
